# Genomic and *in-situ* Transcriptomic Characterization of the Candidate Phylum NPL-UPL2 From Highly Alkaline Highly Reducing Serpentinized Groundwater

**DOI:** 10.3389/fmicb.2018.03141

**Published:** 2018-12-18

**Authors:** Shino Suzuki, Kenneth H. Nealson, Shun’ichi Ishii

**Affiliations:** ^1^Kochi Institute for Core Sample Research, Japan Agency for Marine-Earth Science and Technology, Nankoku, Japan; ^2^Department of Microbial and Environmental Genomics, J. Craig Venter Institute, La Jolla, CA, United States; ^3^Department of Earth Sciences, University of Southern California, Los Angeles, CA, United States; ^4^R&D Center for Submarine Resources, JAMSTEC, Nankoku, Japan

**Keywords:** serpentinization, metagenome, acetogen, last universal common ancestor, alkaliphile ecology, subsurface microbial community, metatranscriptome, carbon monoxide dehydrogenase

## Abstract

Serpentinization is a process whereby water interacts with reduced mantle rock called peridotite to produce a new suite of minerals (e.g., serpentine), a highly alkaline fluid, and hydrogen. In previous reports, we identified abundance of microbes of the candidate phylum NPL-UPA2 in a serpentinization site called The Cedars. Here, we report the first metagenome assembled genome (MAG) of the candidate phylum as well as the *in-situ* gene expression. The MAG of the phylum NPL-UPA2, named Unc8, is only about 1 Mbp and its biosynthetic properties suggest it should be capable of independent growth. In keeping with the highly reducing niche of Unc8, its genome encodes none of the known oxidative stress response genes including superoxide dismutases. With regard to energy metabolism, the MAG of Unc8 encodes all enzymes for Wood-Ljungdahl acetogenesis pathway, a ferredoxin:NAD^+^ oxidoreductase (Rnf) and electron carriers for flavin-based electron bifurcation (Etf, Hdr). Furthermore, the transcriptome of Unc8 in the waters of The Cedars showed enhanced levels of gene expression in the key enzymes of the Wood-Ljungdahl pathway [e.g., Carbon monoxide dehydrogenase /Acetyl-CoA synthase complex (CODH/ACS), Rnf, Acetyl-CoA synthetase (Acd)], which indicated that the Unc8 is an acetogen. However, the MAG of Unc8 encoded no well-known hydrogenase genes, suggesting that the energy metabolism of Unc8 might be focused on CO as the carbon and energy sources for the acetate formation. Given that CO could be supplied via abiotic reaction associated with deep subsurface serpentinization, while available CO_2_ would be at extremely low concentrations in this high pH environment, CO-associated metabolism could provide advantageous approach. The CODH/ACS in Unc8 is a Bacteria/Archaea hybrid type of six-subunit complex and the electron carriers, Etf and Hdr, showed the highest similarity to those in Archaea, suggesting that archaeal methanogenic energy metabolism was incorporated into the bacterial acetogenesis in NPL-UPA2. Given that serpentinization systems are viewed as potential habitats for early life, and that acetogenesis via the Wood-Ljungdahl pathway is proposed as an energy metabolism of Last Universal Common Ancestor, a phylogenetically distinct acetogen from an early earth analog site may provide important insights in primordial lithotrophs and their habitat.

## Introduction

It is an honor to take part in this issue reminding us of the many accomplishments of Professor Koki Horikoshi in the world of alkaliphiles (Horikoshi, 1971, 1996, 1999; Kudo, 2016). We discuss here properties of a member of an undescribed phylum of bacteria that we propose naming after Professor Horikoshi. The metagenome assembled genome (MAG) of the bacteria was recovered from The Cedars, an ancient and widespread environment called a serpentinization site (Schulte et al., 2006; Sleep et al., 2011; Sleep, 2018), where highly alkaline (pH ≥ 11.5) anoxic strongly reducing water (*E*_h_ = -900 to -500 mV) is produced by geological processes (Morrill et al., 2013; Schrenk et al., 2013; Suzuki et al., 2017). The properties we discuss here are inferred from analysis of the gene content of the MAG of Unc8, as well as examination of the *in situ* transcribed genes of Unc8.

Having environmentally relevant microbes in culture is beneficial for microbiology; genomic, transcriptomic and proteomic analyses, when coupled to physiological data can reveal how microorganisms interact with their environment and other microorganisms, thus defining the ecophysiology of the microorganisms and their interactions (Strous et al., 2006; Ettwig et al., 2010; Suzuki et al., 2014; Laso-Perez et al., 2016; McGlynn, 2017; Kato et al., 2018). However, cultivation of environmentally relevant microbes is not always possible, and one is left with the challenge of piecing together the metabolic roles of the microbes using molecular approaches. Improvement of sequencing technologies and the development of bioinformatic techniques have enabled the recovery of high-quality genomes from environmental metagenomes, making it possible to address potential microbial function(s) and roles in the natural ecosystem (Wrighton et al., 2012; Hug et al., 2013; Ishii et al., 2013; Suzuki et al., 2017; Ishii et al., 2018; Probst et al., 2018; Woodcroft et al., 2018).

Recent studies based on MAGs have revealed unprecedented insights into microbial diversity, including the identification of “Candidate Phyla Radiation” (CPR), with entire phyla having significantly reduced genomes that lack many of the genes responsible for biosynthesis and energy metabolism (Brown et al., 2015; Anantharaman et al., 2016; Suzuki et al., 2017; Castelle and Banfield, 2018), and the identification of distributed methane-producing potential in not only the *Euryarchaeota* but in the *Bathyarchaeota* and the *Verstraetearchaeota* (Evans et al., 2015; Vanwonterghem et al., 2016). All those facts have demonstrated that a wealth of evidence of unrecognized microbial diversity may lie buried in the genomes of these uncultivated microorganisms.

We report here the study of the MAG of one of these candidate phyla, NPL-UPA2, in the domain Bacteria. While the initial MAG named Unc8 was retrieved from the metagenome of highly alkaline springs at The Cedars serpentinization site in our previous studies (Morrill et al., 2013; Suzuki et al., 2013, 2017), detail of the metabolic capabilities has not been analyzed. In this study, in order to illustrate the potential metabolic and physiological features of the undescribed phylum NPL-UPA2, we have refined the MAG of Unc8 with additional sequencing and bioinformatics efforts and analyzed the gene expression profile in The Cedars springs. Analyses of the MAG of Unc8 and the transcriptome of natural communities in The Cedars springs suggested that acetogenesis via the Wood-Ljungdahl pathway is the key energy metabolism of this organism. Although serpentinization systems are viewed as potential habitats for early life and the acetogenesis via the Wood-Ljungdahl pathway is proposed as an energy metabolism of primordial lithotrophs, genomic and physiological features of acetogens inhabiting serpentinization sites remain undescribed; thus, the understanding may contribute to idenify the life strategies of primordial acetogens and their habitats.

## Materials and Methods

### Sample Collection

Microbial samples were collected from two different hyperalkaline springs in The Cedars active serpentinization site, BS5sc (elevation 282 m, N: 38°37.282’, W: 123°7.987’) that is a source water of BS5 spring (Suzuki et al., 2013) and Grotto Pool Spring 1 (GPS1) (elevation 273 m, N: 38°37.268’ W: 123°8.014’), by using 0.22 μm in-line filters (Millipore) as described previously (Suzuki et al., 2017). For GPS1 spring, approximately 1000 L of spring water was collected in 2011 and 2012 (Suzuki et al., 2017), while approximately 200 L of spring water was collected for BS5sc in 2014. The filtered cells were immediately frozen with dry ice at each sampling site and kept at dry ice temperature during the transportation. The samples were stored in -80°C in our lab until the DNA and RNA are extracted.

### DNA and RNA Sequencing

Both DNA and RNA were coextracted using a MObio PowerBiofilm RNA Isolation Kit (MO BIO, San Diego, CA, United States) as described previously (Suzuki et al., 2017). The extracted total nucleic acids were eluted in nuclease free water and separated into DNA and RNA using AllPrep DNA/RNA Mini Kit (Qiagen, Germantown, MD, United States). A DNA library of the GPS1 sample was prepared and sequenced as described previously (Suzuki et al., 2017). A DNA library of the BS5sc sample for NGS was prepared from 1 ng DNA using the Nextera XT library preparation Kit (Illumina, San Diego, CA, United States) according to the manufacturer’s protocol. Total RNAs from both GPS1 and BS5sc samples were treated with Turbo DNA free kit (Thermo Fisher Scientific, Waltham, MA, United States) for the complete removal of contaminating DNA. DNase-treated total RNA samples were directly applied for library construction by using ScriptSeq v2 (Illumina, San Diego, CA, United States) without rRNA removal step to avoid unnecessarily bias.

The DNAs were separately sequenced using Illumina HiSeq2000 platform (Illumina, San Diego, CA, United States) as the 101 bp PE for GPS1 samples and as the 151 bp PE for BS5sc samples by Illumina’s standard protocol. The DNA sequences of GPS1 sample have already been deposited in the NCBI Short Read Archive (SRA) under accession numbers DRX086601 and DRX086602, while newly sequenced metagenomic reads from the BS5sc sample was deposited in the SRA under accession number SRX5014375. RNAs from GPS1 and BS5sc samples were sequenced using Illumina HiSeq2000 platform (Illumina, San Diego, CA, United States) as the 101 bp PE for GPS1 sample and as the 151 bp PE for BS5sc sample by Illumina’s standard protocol. Read stats of DNA and RNA sequences are shown in Supplementary Table S1.

Metagenomic reads from biofilm of hydrothermal field in Prony Bay (SRA; SRS734862 and SRS734863) were used for *de novo* assembly of CLC Genomic Workbench v8.6 (CLCbio, Boston, MA, United States) with default parameters.

### Genome Refinement of Unc8

A MAG of NPL-UPA2 bacterium Unc8, recovered from the GPS1-2012 metagenome (Suzuki et al., 2017), was used as a template for the further genome refinement in this study. The contaminated scaffolds in the MAG were removed by using differential coverage plots between GPS1 metagenomes and the new BS5sc metagenome (Albertsen et al., 2013; Ishii et al., 2013). The cross-read mapping analyses were run using Map Reads to Reference algorism in CLC Genomics Workbench (version 8.5) with the settings as 0.7 of minimum length and 0.95 of minimum similarity fractions. The scaffolds of MAG Unc8 were then cleaved to contigs at the gap regions. The potential connections of contigs were analyzed by Collect Paired Read Statistics tool in CLC Genome Finishing Module (CLCbio, Boston, MA, United States). The analysis allowed to remove wrong contigs included in the MAG Unc8 (Albertsen et al., 2013). Based on the potential connections, the contigs were manually connected by using Align Contigs tool after the extension of contig edge by using Extend Contig tool in CLC Genome Finishing Module. After the manual curation, in order to polish contigs, metagenomic reads of GPS1 2011 and 2012 were mapped to the contigs with the settings as 0.7 of minimum length and 0.95 of minimum similarity fractions, and the consensus sequences were extracted. The refined MAG Unc8 was deposited in NCBI under Biosample SAMN06718453.

To obtain the minimum information about a MAG (miMAG) proposed by Genomic Standards Consortium (Bowers et al., 2017), genome completeness and contamination were analyzed by using CheckM software (Parks et al., 2015) on KBase (Arkin et al., 2018). The numbers of tRNA and rRNA were counted by using NCBI prokaryotic genome annotation pipeline (Tatusova et al., 2016). The genome quality classification was assigned from miMAG criteria (Bowers et al., 2017).

A BLAST Ring image generator (BRIG) (Alikhan et al., 2011) was employed for visualizing a genome as a circular image and for comparison between the MAG Unc8 from The Cedars spring and the metagenomic contigs of ST09 from Prony Bay hydrothermal field (Mei et al., 2016). The total DNA and RNA reads of BS5sc spring were separately mapped to the Unc8 contigs by using CLC Genomics Workbench with the settings as 0.5 of minimum length and 0.95 of minimum similarity fractions. From the SAM files of the read mapping, coverage graph was generated in the BRIG software, and the coverage graph of the RNA reads were normalized by the coverage graph of the DNA reads.

### Functional Annotation

Metagenome assembled genome of Unc8 was processed in NCBI prokaryotic genome annotation pipeline for open reading frame (ORF) calling and functional annotation (Tatusova et al., 2016). For the KEGG orthologous (KO) group assignment for each ORF, we used the KEGG Automatic Annotation Server (KAAS) with the SBH (single-directional best hit) method set to 37 as the threshold assignment score (Moriya et al., 2007). ORFs were assigned to the Clusters of Orthologous Groups of proteins (COGs) by the best BLAST hit to the reference data (Galperin et al., 2015) using an *e*-value cutoff of 1*e*^-6^. Localization of the proteins was analyzed by prediction of transmembrane helices in TMHMM server version 2.0 (Krogh et al., 2001), and PSORTb version 3.0.2 (Yu et al., 2010). Taxonomic assignment of each ORF was analyzed by using GhostKOALA (Kanehisa et al., 2016). Microbial cell activity-, biogenesis, and metabolisms-associated marker genes were selected from the KEGG module or KEGG pathway databases and analyzed as described previously (Ishii et al., 2015; Suzuki et al., 2017; Ishii et al., 2018). Protein abbreviations used in this study are summarized in Supplementary Table S2.

### Read Mapping of Raw Reads to ORFs

RPKM (Reads Per Kilobase per Million mapped reads) values (Mortazavi et al., 2008) for both DNA and mRNA samples were separately generated by the RNA-Seq Analysis pipeline in CLC Genomics Workbench (version 8.6), and used for analyzing ORF frequency (DNA-RPKM) and gene expression levels (mRNA-RPKM). The Unc8 ORFs were used as references, and read mapping was conducted using 0.5 as the minimum length and 0.95 as the minimum similarity fractions. The calculated median of the DNA-RPKM for each sample was used to normalize the related mRNA-RPKM values for each sample.

### Phylogenetic Tree Analyses

Amino acid sequences of CdhA, CdhB, CdhC, CdhE/AcsC, CedD/AcsD, AcsE in the Unc8 MAG were blasted against nr database. Twenty closest amino acid sequences were retrieved from the database for applying the tree construction. MUSCLE (Edgar, 2004) and Maximum Likelihood with RaxML (Stamatakis et al., 2008) were used for the sequence alignment and tree construction, respectively.

## Results and Discussion

### Genome Quality of the MAG of the Cedars NPL-UPA2

In our previous study, the MAG of Unc8 was recovered from the two different metagenomic assemblies delivered from the two different years’ samples (2011 and 2012) of GPS1 at The Cedars (Suzuki et al., 2017). The MAG of Unc8 was constituted with 166 scaffolds and 291 contigs. In this study, the MAG was further refined with the assembled data of the other spring (BS5sc), and the genome size now became 996,215 bp consisting of only 24 contigs (Table 1 and Supplementary Figure S1). Genome completeness was 87.6% estimated by the Check M with the Bacterial marker linkage (Parks et al., 2015). MAG criteria for the high-quality genome are (1) over 90% of the completeness, (2) a less than 5% contamination, (3) multiple fragments where gaps span repetitive regions, (4) the presence of the 23S, 16S and 5S rRNA genes and (5) at least 18 tRNAs. The MAG of Unc8 meets the criteria except for that of over 90% completeness (87.6%). Thus, the MAG of Unc8 is a middle-quality draft genome (Table 1). However, it is likely that the NPL-UPA2 has a genome lacking a number of the single copy marker genes listed in the bacterial marker linkage from the genome as is often seen in the genomes of other candidate divisions (Suzuki et al., 2017), and if this is the case, the quality should be very close to that of a high-quality draft genome, and the MAG of Unc8 may well be appropriate for further genomic and transcriptomic investigation.

**Table 1:** MAG criteria and genome stats for Unc8.

**General genome metadata currently not in MIGS**	
Analysis project type	Metagenome-assembled genome (MAG)
Taxa id	16S rRNA gene (KC574886)
Assembly software	CLC Genomics Workbench 8.6
Annotation	NCBI Prokaryotic Genome Annotation Pipeline (PGAP)
**Genome quality**	
Assembly quality	Nearly High-Quality Draft
Completeness score	87.60%
Contamination score	2.43%
Completeness software	checkm
Number of contigs	24
16S recovered	Yes
16S recovery software	rnammer
Number of standard tRNAs extracted	20
tRNA extraction software	trnascan-se
Completeness approach	Marker gene based
**MAG metadata**	
Bin parameters	Coverage + broken pair mates
Binning software	Differential Coverage plots (by hand)
	CLC Genome Finishing Module
Reassembly post binning	Yes
MAG coverage software	CLC Genomics Workbench 8.6
**Genome stats**	
Genome size	996,215 bp
Number of contigs	24
N50	77,401 bp
Frequency in GPS1 (2011)	×34.6 (0.9%)
Frequency in GPS1 (2012)	×31.1 (0.8%)
Frequency in BS5sc (2014)	×48.1 (0.8%)
BioProject	PRJNA351917
BioSample	SAMN06718453
CDS (coding)	985
Complete rRNAs	1, 1, 1 (5S, 16S, 23S)
tRNAs	43
ncRNAs	2
Pseudo Genes (total)	18
Potential membrane associated proteins	181


### Phylogeny and the Environmental Distribution of NPL-UPA2

**FIGURE 1:**
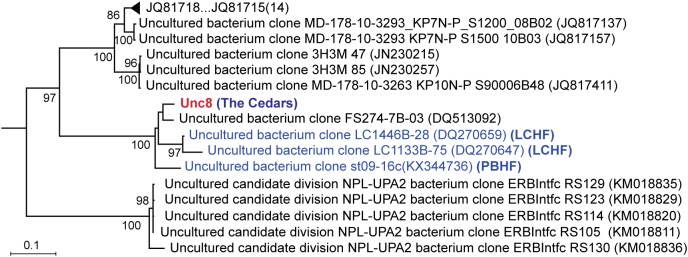
Phylogenetic relationships among NPL-UPA2 16S rRNA genes. Tree topologies are supported by bootstrap values for 100 replicates. Red font denotes the Unc8 from The Cedars and blue font denotes phylotypes recovered from the serpentinization sites; Lost City hydrothermal field (LCHF) and Prony Bay hydrothermal field (PBHF). Accession numbers of OTU included in the triangle region are JQ816227, GU553753, JQ817159, JN676031, JX000976, JQ817187, JQ817075, JQ816462, JQ817138, JQ816442, JN230011, EU385719, JQ816456 and JQ818038. Ca. *Desulforudis audaxviator* was root of the tree.

Members of the candidate phylum NPL-UPA2 have been detected in a variety of different environments, including the oceanic subsurface sediment (Hoshino et al., 2011), deep-sea anoxic brines (Guan et al., 2015), crustal fluids (Huber et al., 2006) and subterranean serpentinization sites (Brazelton et al., 2006; Postec et al., 2015) (Figure 1). The 16S rRNA genes recovered from the three serpentinization sites, Lost City (Brazelton et al., 2006), Prony Bay (Postec et al., 2015) and The Cedars (Suzuki et al., 2013) group together as a clade in the phylogenetic tree. In general, members of this phylum were detected as rare members of the respective communities. Relatively abundant populations of NPL-UPA2 have been reported only at the shallow marine serpentinizing Prony Hydrothermal Field (13.8%) (Postec et al., 2015) and the deep groundwater of the continental serpentinizing site The Cedars (4%) (Suzuki et al., 2013).

Geochemical studies of The Cedars springs revealed that the site has two different serpentinized water sources, a deep source that interacts with peridotite body as well as km-deep marine sediments, and a shallow source that interacts only with the overlying peridotite (Morrill et al., 2013; Suzuki et al., 2013). Considering that the Unc8 is associated with The Cedars deep groundwater which is influenced by the subducted oceanic plate below the peridotite body, and that other members of this phylum are also associated with marine subsurface environments (Brazelton et al., 2006; Huber et al., 2006; Hoshino et al., 2011; Guan et al., 2015; Postec et al., 2015), it may well be that such marine, subsurface, anoxic, highly reducing environments define the habitat of the phylum NPL-UPA2.

### Habitat of Unc8 and Its Closest Relative in the Phylum NPL-UPA2

The MAG of Unc8 within the phylum NPL-UPA2 was recovered from the metagenomic sequences of The Cedars serpentinized spring and further refined in this study. Serpentinization is a process whereby water interacts with ultramafic minerals (e.g., peridotite) delivered from the Earth’s mantle to produce a new suite of rock (e.g., serpentinite) (Schrenk et al., 2013). The reaction results in the oxidation of ferrous iron from olivine and pyroxene minerals in the peridotite with molecular hydrogen being produced during the oxidation process. The hydrogen and carbon dioxide present in the system are thought to react under the highly reducing and alkaline conditions through Fischer-Tropsch Type (FTT) synthesis, leading to the formation of methane and hydrocarbons and the concomitant production of carbon monoxide, formate, formaldehyde and methanol (McCollom and Seewald, 2001; McCollom and Seewald, 2007; Schrenk et al., 2013). Since the reduced compounds in the fluid can support microbial energy metabolisms, an energy-rich fluid containing organic carbon could be a favorable habitat for life. However, studies of deep fluids in serpentinized setting have shown that these ecosystems host extremely low-abundance microbial communities (Brazelton et al., 2012; Suzuki et al., 2013; Tiago and Verissimo, 2013), which is attributed to: (1) the highly alkaline condition of the fluid; (2) the extremely low concentrations of oxidants (electron acceptors); and, (3) the low levels of nutrients (available carbon and phosphate).

The Cedars is an active terrestrial serpentinization site located in northern California (Morrill et al., 2013). While there are about a hundred of springs in The Cedars area with a variety of differences in geochemistry, spring waters discharged from The Cedars generally have extremely high pH (11–12), very low *E*_h_ (-900—550 mV) values and are rich in Ca^2+^ (∼1 mM), hydrogen and methane gas, and contain low levels of dissolved organic carbon, total inorganic carbon, ammonium, phosphate and electron acceptors (oxygen, nitrate, sulfate) (Morrill et al., 2013; Suzuki et al., 2013).

**FIGURE 2:**
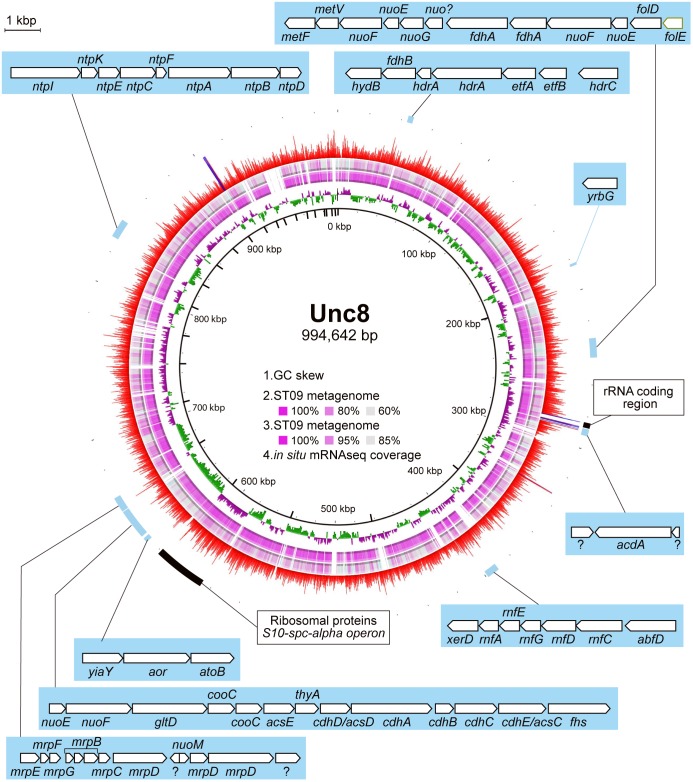
MAG of Unc8 with key genes highlighted. The inner scales designate the coordinates (in kbp). The first (inner-most) circle shows the GC-skew. The second and third circles show the blastn analysis against the Prony Bay metagenome with different similarity cutoff. The lower and upper identity threshold in the blastn analysis is shown in the circle. The fourth circle shows the gene expression level (RNA reads normalized by the DNA reads) in BS5sc. The locations of some genes of interest are indicated.

Comparison of the contigs assembled from the Prony Bay metagenome (Mei et al., 2016) revealed high similarity to the MAG of Unc8, suggesting that Unc8-like microbe(s) are present in the Prony Bay Hydrothermal Field and may share similar evolutionary histories with Unc8 (Figure 2). The Prony Bay Hydrothermal Field is also an active site of serpentinization but at the seafloor in a shallower lagoonal environment (Monnin et al., 2014). Fluids discharged from the Prony Bay are the high-pH fluids (pH = ∼10.5) rich in H_2_ and CH_4_. While the outlet of the fluid is located in the seafloor, the high-pH fluid is of meteoric origin.

### Biosynthesis, Stress Response, Motility, Transporters and Thermophily of the Unc8

**FIGURE 3:**
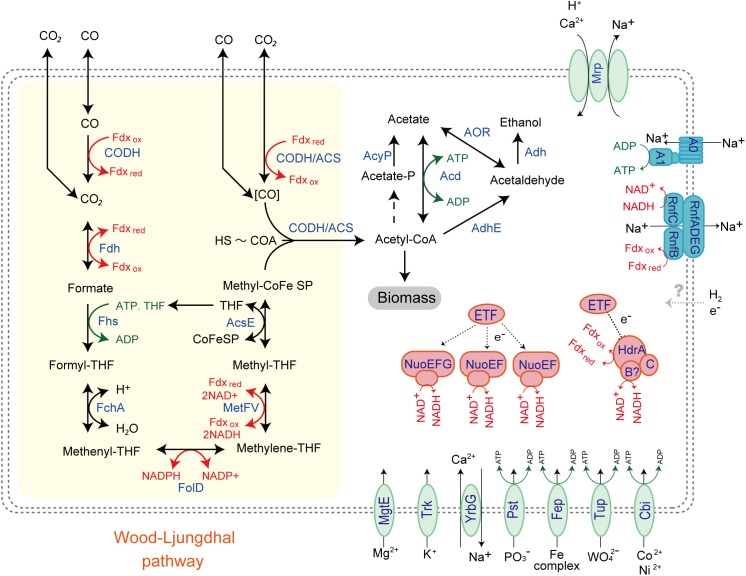
Predicted energy metabolism for Unc8. Proteins and complexes are colored based on the functional categories. Abbreviations: CODH-Carbon monoxide dehydrogenase, Fdh-Formate dehydrogenase, Fhs-Formyl-THF synthase, Fch-Formyl-THF cyclohydrolase, FolD-Methylene-THF dehydrogenase, MetFV-Methylene-THF reductase, AcsE-Methyltransferase, CODH/ACS-Carbon monoxide dehydrogenase/Acetyl-CoA synthase complex, AcyP-Acylphosphatase, Acd-ADP forming Acetyl-CoA synthetase, Adh-Alcohol dehydrogenase, Nuo-NADH-quinone oxidoreductase, ETF-Electron transfer flavoprotein, HdrABC-Heterodisulfide reductase, Mrp-Multiple resistance and pH antiporter, A_1_A_O_-ATPase,-an Archaeal type ATPase, Rnf-proton/sodium-translocating ferredoxin-NAD:oxidoreductase complex, MgtE-Magnesium transporter, Trk-Potassium uptake system, YrbG-Ca^2+^/Na^+^ antiporter, Pst-ABC-type Phosphate transporter, Fep-Iron transporter, Tup-Tungstate transporter, Cbi-Cobalt/Nickel transporter.

**FIGURE 4:**
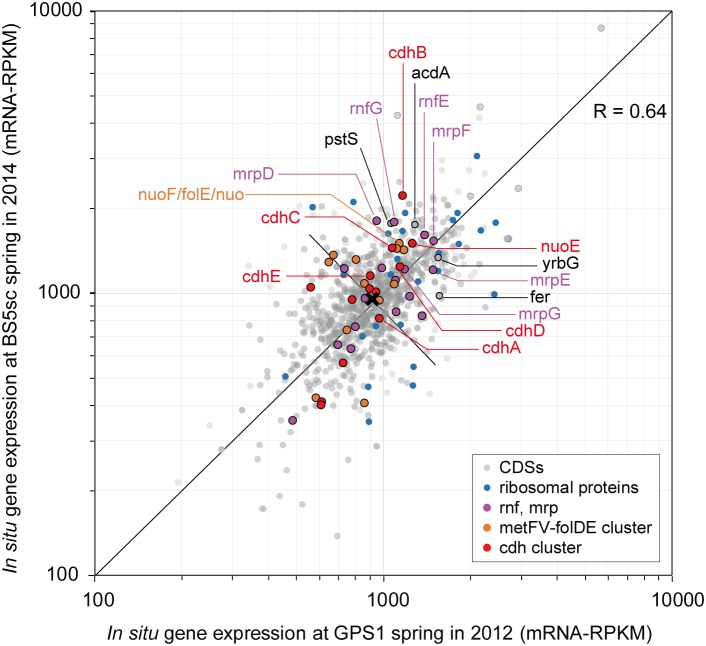
Scatter plots of *in situ* gene expression of Unc8 in The Cedars springs. Scatter plots of all CDS in Unc8 responses as measured by mRNA frequency (mRNA-RPKM) between GPS1 and BS5sc. Key genes for acetogenesis are shown as unique colors, all other groups are shown in gray.

While the MAG size of Unc8 is small, only about 1 Mbp, it encodes complete biosynthetic pathways for amino acids, nucleic acids, lipids, lipopolysaccharide and peptidoglycan, suggesting that Unc8 is capable of living independently (Supplementary Data S2B). Diverse inorganic ion transporters are also encoded (Supplementary Data S1), including the ABC type phosphate (PstABCS), iron (FepBDC), tungstate (TupABC) and cobalt/nickel (CbiOQML) transporters, Ca^2+^/Na^+^ antiporter (YrbG), potassium uptake system (TrkAH), magnesium transporter (MgtE) and multisubunit Na^+^/H^+^ antiporter complex (MrpEFGBBBCD, MrpDD) (Figure 3). Protein abbreviations are summarized in Supplementary Table S2. Since the Mrp complex is involved with the maintenance and homeostasis of the cytosolic pH (Ito et al., 2017), one expects it to be important for life in the highly alkaline environment. High level of expression was seen in the genes for the YrbG (Besserer et al., 2012) and PstS (Liu et al., 1998) in both springs, implying that Unc8 is managing against the extremely alkaline and low phosphate condition occurring at the setting (Figure 4). Other than the inorganic ion transporting system, the MAG of Unc8 encodes only three other transport systems (basic amino acid/polyamine antiporter, biopolymer transport protein, glycoside/pentoside/hexuronide:cation symporter) This paucity of transporters is curious, but may suggest that the Unc8 is incapable of importing organic/inorganic carbon from the environment via transporters. Except for genes coding for type VI pili, no motility-related genes (flagellum chemotaxis) were encoded (Supplementary Data S1).

Notably, oxidative stress response genes (catalase and superoxide dismutase) are absent on the genome of Unc8 (oxidative stress section in Supplementary Data S2A). Since many anaerobic bacteria and archaea are known to have superoxide dismutases to provide protection from radical oxygen species (ROS), and even the Last Universal Common Ancestor (LUCA) is also estimated to encode the enzymes (Slesak et al., 2012; Weiss et al., 2016), the lack of these suggests that Unc8 and perhaps the NPL-UPA2 relatives are relegated to very reduced environments on the Earth; whether this has any impact on our view of LUCA remains an interesting question.

Given that serpentinization is an exothermic process, and that the geothermal gradient leads to the subsurface warming with depth, the habitat of Unc8 in the deep ground water is almost certainly at higher temperature than that encountered in surface environments. Zeldovich et al. (2007) reported that fraction of a set of amino acids, namely isoleucine, valine, tryptophan, arginine, glutamic acid, and leucine, in whole coded proteins is highly correlated with the optimum temperature for growth of every organism. The enumerated quantitative relationship between the optimum growth temperature (*T*_opt_) and fraction F of IVYWREL amino acids reads estimated that the optimum growth temperature of the Unc8 was 67.36°C. Meanwhile, G+C contents of the 16S rRNA gene is also reported the strong correlation to the optimum growth temperatures of prokaryote (Kimura et al., 2006). Estimation of optimum growth temperature of the Unc8 based on the G+C content of 16S rRNA gene was 43.09°C. Both results suggest that habitat of Unc8 should be high: perhaps it is a thermophile. This also remains an interesting question: one whose answer may depend on obtaining an Unc8 cultivar.

### Energy Metabolism of the Unc8

The MAG of Unc8 encodes limited metabolic potentials (Figure 3 and Supplementary Data S2C). It does not encode genes for the TCA cycle, terminal electron acceptor reductases (cytochrome oxidase, sulfate reductase and nitrate reductase) or a standard electron transport chain including cytochromes and all membrane-bound Complex I subunits, indicating that Unc8 does not possess a typical respiratory metabolism. Furthermore, while a nearly complete set of glycolysis pathway genes is present, neither glucose transporters nor the genes responsible for forming glucose-6-phospate from glucose are seen. Since the origin of the deep water at The Cedars is far removed from the photosynthetic world, fermentative metabolism of sugars is not expected. In keeping with this, the genes responsible for the glycolysis pathway exhibited very low expression levels in the transcriptomic analyses (Supplementary Data S1). Amino acid fermentation is also unlikely to occur in the Unc8 because the MAG of Unc8 encodes no ABC-type amino acid transport systems to uptake amino acids from the outside of the cell (Supplementary Data S2C). As mentioned above, the MAG of Unc8 encodes almost no transporters to uptake organic compounds from outside of the cell, thus, substrates for the energy metabolisms of Unc8 must be permeable molecules, probably dissolved gasses or perhaps low molecule compounds that are transportable without specific transporters.

**FIGURE 5:**
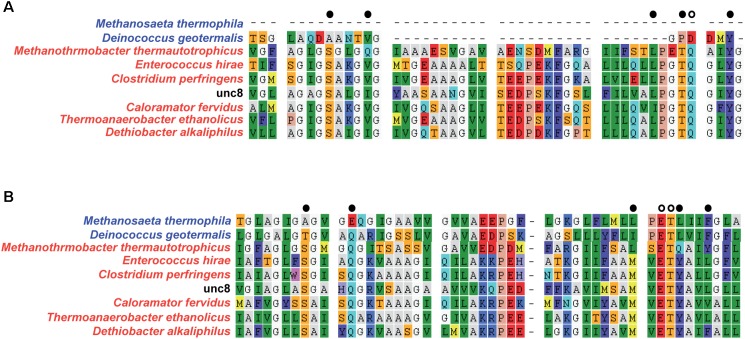
Alignment of NtpC amino acid sequences from Unc8 to determine the interactive cations (i.e., sodium or proton) (Mulkidjanian et al., 2008). N-end **(A)** and C-end **(B)** of NtpC that include active sites are shown. Colors in the species names indicate the genomes encoding proton (blue) or sodium (red) coupled A-ATPases. Active site residues are shown with black/white dots at the top of each alignment and white dots indicate specific residues that are conserved in sodium-ligands and are partly absent in proton binding subunit (Mulkidjanian et al., 2008).

Genomic and transcriptomic data suggest that the major metabolism of the Unc8 is acetogenesis presumably involved in the Wood-Ljungdahl pathway which is the only pathway that couples the fixation of inorganic carbon to energy conservation (Schuchmann and Muller, 2014) (Figure 3 and Supplementary Data S1 and S2C). The MAG of Unc8 encodes key enzymes for the Wood-Ljungdahl pathway including Carbon monoxide dehydrogenase/Acetyl-CoA synthase complex (CODH/ACS), Formate dehydrogenase (Fdh), Formyl-THF synthase (Fhs), Formyl-THF cyclohydrolase (Fch), Methylene-THF dehydrogenase (FolD), Methylene-THF reductase (MetVF), Methyltransferase (AcsE). The ATP synthase of Unc8 is an Archaeal type ATPase (A-ATPase), and the homology search of NtpC, a c-subunit of A_1_A_O_-ATPase, indicated a sodium-dependent A-ATPase (Figure 5). The MAG of Unc8 harbors genes for the proton/sodium-translocating ferredoxin-NAD:oxidoreductase complex (Rnf) (Biegel et al., 2011; Buckel and Thauer, 2013, 2018), which is presumably a key complex for the sodium translocation from the cytosol to the cell exterior to power the sodium-dependent A-ATPase for the ATP production (Mulkidjanian et al., 2008; Chowdhury et al., 2016). YrbG (Ca^2+^/Na^+^ antiporter) (Besserer et al., 2012), MrpEFGBBBCD and MrpDD (Na^+^, Ca^2+^, K^+^/H^+^ antiporter) (Ito et al., 2017) also may contribute to the export of sodium for ATP production. Three sets of *nuo* gene cluster (two sets of *nuoEF* and one set of *nuoEGF*), which encode a NADH dehydrogenase module, are also present on the genome and those may serve to regenerate NADH. Sets of the *nuoEF* and *nuoEGF* are located close to the *fdhAB* (Formate dehydrogenase) and *folD* (Methenyl-THF cyclohydrolase) on the genome and one set of the *nuoEF* is close to the gene cluster coding the CODH/ACS complex (Figures 2, 3). Such close localizations of related genes on the genome may indicate that those are controlled under the same regulatory system. The MAG of Unc8 also contains the genes coding for the cytoplasmic electron transfer proteins through a flavin-based electron bifurcation mechanism, including two sets of the EtfAB (Electron transfer flavoprotein) and one set of HdrABC-like complex (Heterodisulfide reductase) (Buckel and Thauer, 2013, 2018). While genes for the HdrA and HdrC were identified, one for HdrB was not present. Since the heterodisulfide of coenzyme M and coenzyme B (CoM-S-S-CoB), a substrate of HdrB in typical methanogenic archaea, is not present in bacterial acetogens, the feature is reasonable. Based on the gene locations, HdrB in Unc8 was replaced with HydB (Sulfhydrogenase beta subunit) which is also a reductase with 4Fe-4S cluster, but the substrate is NADH/NAD^+^. One set of the *etfAB* genes and the *hydB-hdrAC* genes are located in tandem on the MAG (Figure 2), implying that the Hdr-like complex works together with Etf instead of Mvh, a hydrogenase coupled with Hdr complex in methanogens (Buckel and Thauer, 2018). Further investigations are required.

The end products of Unc8 could be either acetate or ethanol as evidenced by the presence of Acd and alcohol dehydrogenase (AdhE, Adh) on the genome (Figure 3 and Supplementary Data S2C). Higher expression was seen in the gene for Acetyl-CoA synthetase that is capable of converting Acetyl-coA to acetate with the formation of ATP (Musfeldt and Schonheit, 2002) (Figure 4), implying that the acetogenesis is the major mode for the energy conservation in the Unc8.

Taken together, these data suggested that Unc8 employs acetogenesis for energy conservation. The gene expression profile is consisting with this idea, showing the importance of the Ferredoxin, CODH/ACS, ADP forming Acetyl-CoA synthetase and Rnf complex as indicated by the higher expression of genes (Figure 4). However, the absence of any of well-known hydrogenases genes on the genome is puzzling (see Section “Hydrogenase” in Supplementary Data S2C): Hydrogenases catalyze the oxidation of hydrogen and allow bacteria to use hydrogen as an energy source for their growth. All the cultivated acetogens with the Wood-Ljungdahl pathway are able to gain energy through hydrogen-oxidizing CO_2_-reducing acetate formation and the reaction is generally described as 4 H_2_ + 2 CO_2_ → CH_3_COOH + 2 H_2_O (ΔG^0^ = -95 kJ/mol). In addition, all of the cultivated acetogens known so far encode genes for hydrogenases on their genomes (Schuchmann and Muller, 2012) and hydrogen is the most abundant reduced substrate in the serpentinized fluid from The Cedars (Morrill et al., 2013). One possibility for unusual absence of hydrogenase genes is that Unc8 has uncharacterized hydrogenases coded by the hypothetical or function-unknown genes. Alternatively, it is possible that the Unc8 does not employ hydrogen-oxidizing CO_2_-reduction but rather exploits carbon monoxide for acetate formation and energy conservation (Bertsch and Muller, 2015) as described 4 CO + 2 H_2_O→ CH_3_COOH + 2 CO_2_ (ΔG^0^ = -165.5 kJ/mol). If carbon monoxide is available, utilization of CO would have a significant advantage in this environment due to the CO_2_, a key substrate for acetogenesis, which will be present at extremely low concentrations because of the high innate alkalinity and the high concentration of calcium and as the potential energetical advantage (Diender et al., 2015). While organisms that can grow with only CO are rare, a few are known *Thermoanaerobacter kivui* (Weghoff and Muller, 2016) and *Methanosarcina acetivorans* C2A (Rother and Metcalf, 2004) in which CO consumption is coupled to acetate formation. Considering that CO is likely being produced as the intermediate of FTT synthesis from CO_2_ to CH_4_ under extremely reducing condition (Schrenk et al., 2013), it is reasonable to suggest that Unc8 employs CO for energy conservation rather than using molecular hydrogen as an electron source for CO_2_ reduction in the deep subsurface. Yet, another possibility is that Unc8 is tightly associated with the reduced minerals or the reduced settings existing in The Cedars (the deep groundwater *E*_h_ is between -900 and -700 mV) and the oxidized ferredoxin or NAD^+^ in the cell are reduced by electrons accumulated outside of the cells via chemical, enzymatic or metal-proteinous reactions.

### Phylogenetic Analysis of Carbon Monoxide Dehydrogenase/Acetyl-CoA Synthase

**FIGURE 6:**
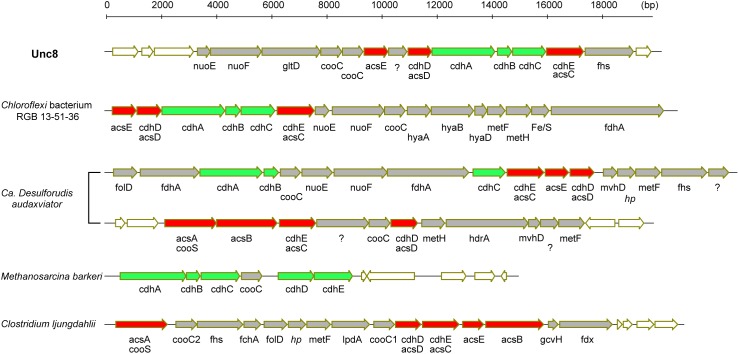
Comparison of gene clusters for CODH and ACS. Green colored genes are archaeal in origin and red colored genes are bacterial in origin. In general, CODH/ACS is a five-subunits enzyme but Unc8, *Chloroflexi* bacterium RGB 13-51-36 and *Ca.* *Desulforudis audaxviator* encode a bacterial and archaeal hybrid type of six-subunits enzyme. *Ca. Desulforudis audaxviator* have two different clusters of gene sets for CODH/ACS on the MAG. Respective homologs of *cdhA, cdhC, cdhD*, and *cdhE* in Archaea are *acsA, acsB, acsD and acsC* in Bacteria (Adam et al., 2018). *cdhB* is exclusive to Archaea and *acsE* is exclusive to Bacteria.

**FIGURE 7:**
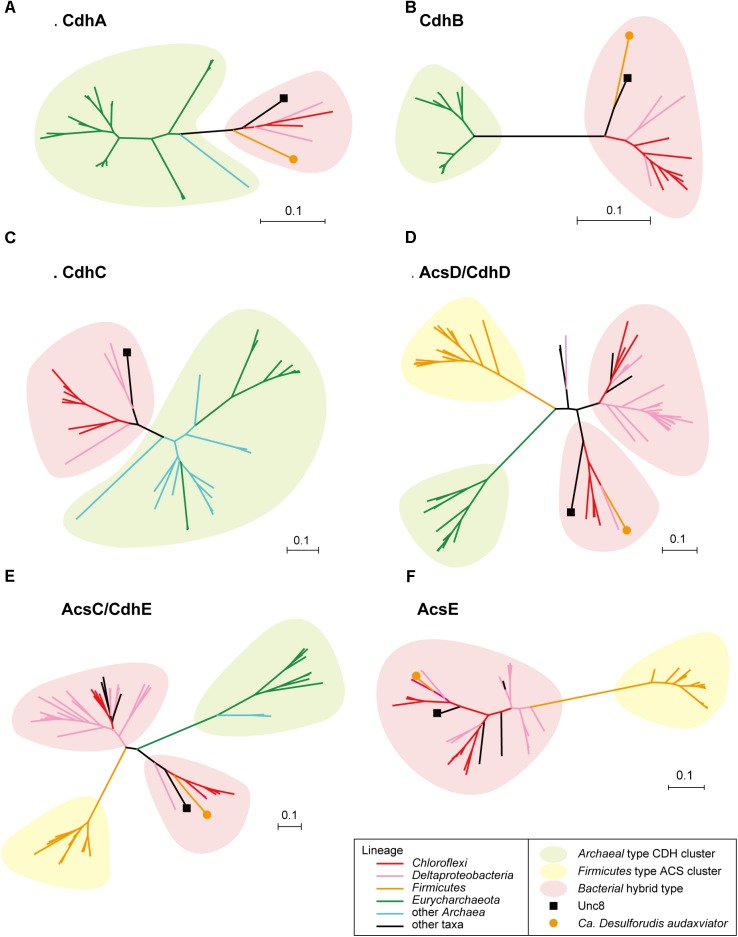
Protein phylogeny of CODH/ACS complex. Phylogenetic trees were constructed for CdhA **(A)**, CdhB **(B)**, CdhC **(C)**, AcsD/CdhD **(D)**, AcsC/CdhE **(E)** and AcsE **(F)**. Detailed trees are shown in Supplementary Figure S2.

Bifunctional CODH/ACS is generally a five-subunit enzyme complex and a key to carbon fixation in the Wood-Ljungdahl pathway (Figure 6). Four of the five subunits are homologous between Bacteria and Archaea. In Archaea, they are called CdhA (α-subunit), CdhC (β-subunit), CdhD (δ subunit), and CdhE (γ subunit), while in Bacteria, their respective homologs are called AcsA (β), AcsB (α), AcsD (δ), and AcsC (γ) (Adam et al., 2018). In addition, there exists a subunit exclusive to Archaea called CdhB (𝜀-subunit), and one exclusive to Bacteria (AcsE) (Figures 2, 6). CdhABC in the Archaea and AcsAB in the Bacteria are responsible for the oxidoreductase module of the CODH/ACS and CdhDE in Archaea and AcsCDE in Bacteria are for the methyltransferase module. CODH/ACS complex in the Unc8 was, however, a hybrid of bacterial and archaeal type, and the CdhABC (oxidoreductase module) is the Archaeal type and AcsCDE (methyltransferase module) is the Bacterial type (Figure 6 and Supplementary Figure S2). Bacteria/Archaea hybrids of six-subunit CODH/ACS are unusual but they have been seen in the MAG of *Candidatus Desulforudis audaxviator* recovered from alkaline groundwater in the deep subsurface gold mine (Chivian et al., 2008) and the MAG of *Chloroflexi* bacterium RGB_13_51_36 recovered from a sediment core drilled from a well at the Rifle Integrated Field Research Challenge (Hug et al., 2013) (Figure 6 and Supplementary Data S3). Although Unc8, *Ca. D. audaxviator* and *Chloroflexi* bacterium RGB_13_51 are affiliated with different phyla (NPL-UPA2, *Firmicutes* or *Chloroflexi*, respectively), our phylogenetic analysis of the individual proteins suggests that Archaeal CdhABC complex was delivered at a single horizontal transfer from the *Euryarchaeota* to the *Bacteria* and that subsequent transfers occurred among bacterial lineages as discussed by Adam et al. (2018) (Figure 6). Phylogenetic trees of CdhA, CdhB and CdhC showed that those enzymes coded by Unc8 are closest to the archaeal cluster among those coded by the other bacterial members, implying that the *cdhABC* gene cluster on Unc8 genome were perhaps delivered from Archaea at the early stage of the horizontal gene transfer (Figure 7 and Supplementary Figure S2). Further genomic studies targeted to other subsurface environments may identify the distribution and evolution of this type of enzyme complex in diverse bacterial phyla (Figure 7 and Supplementary Figure S2). Unfortunately, since there are no cultivated organisms having the hybrids of six-subunit CODH/ACS, the advantages of such enzymes are unclear. The advantages may be related to the life strategies living in the environment depleted carbon dioxide and/or of the carbon monoxide mode of acetogenesis. Further enzymatic biochemistry of the hybrid CODH/ACS may provide us with important insights into the energy metabolism of the subsurface microbes and communities.

### Archaeal Methanogenic Components in Unc8

The MAG of Unc8 indicates that the pathway to acetogenesis involves a mixture of archaeal and bacterial components (Figure 6 and Supplementary Figure S2): while the Wood-Ljungdahl pathway and Rnf are known in association with bacterial acetogenesis (Ragsdale and Pierce, 2008; Schuchmann and Muller, 2014; Buckel and Thauer, 2018), the ATP synthase in Unc8 is an archaeal type (A_1_A_O_), the CODH/ACS is a bacterial/archaeal hybrid type of enzyme (Buckel and Thauer, 2018) and an Hdr complex is usually employed by the methanogenic archaea as an electron carrier. The origin of the electron transfer flavoprotein (EtfAB) is unsure; one of the two sets of *etf*AB genes is located close together with *hydB-hdr*AC genes on the Unc8 genome (Figure 2) and the *etf*AB genes show the highest similarity to those in the *Candidatus* Bathyarchaeota archaeon BA2 (Evans et al., 2015). Similar features were seen in the *Chloroflexi* bacterium RGB_13_51, namely, the *Chloroflexi* bacterium RGB_13_51 partly utilizes archaeal methanogenic system such as A-ATPase, hybrid CODH/ACS and Hdr although the RGB_13_51 is capable of using hydrogenases, one of which (Mvh) is also the Archaeal type of hydrogenase (Hug et al., 2013) (Supplementary Data S3). One interpretation of this is that archaeal methanogenic energy metabolism was incorporated into a bacterial acetogen and then transferred horizontally among bacterial lineages of lithotrophic acetogens.

### Implications

Given that this is the first genomic and transcriptomic description of candidate phylum NPL-UPA2, to honor Professor Horikoshi, we propose the provisional taxonomic assignment to “*Candidatus* Horikoshi bacteria” phylum. nov.. The “*Ca.* Horikoshi bacteria” bacterium Unc8 from highly alkaline highly reducing groundwater at The Cedars is presumably an acetogen via Wood-Ljungdahl pathway. Several of the key enzymes are archaeal in origin. While lack of hydrogenases is puzzling, acetogenesis from carbon monoxide could be a favorable energy metabolism in this setting. Alternatively, during the evolution under highly alkaline and highly reducing condition, Unc8 might obtain some unknown metabolic systems for utilizing reducing power outside of the cell without using well-known hydrogenases and cytochromes. Namely, redox potentials needed to reduce ferredoxin and NADH are *E*_h_ = -430 and -320 mV, respectively (Schuchmann and Muller, 2014). *E*_h_ of The Cedars deep ground water is between –900 and –700 mV at pH 12, which at pH 8 (assumed intracellular pH) would be equivalent to –640 and –440 mV, respectively. If Unc8 can reduce ferredoxin and NADH by using the reducing power outside of the cell in some ways, synthesis of ATP could easily occur by subsequent acetogenesis. Further investigations may shed light on this fascinating question.

As a final point, serpentinizing systems are viewed as both analogs for planetary bodies and potential early Earth environments (Schulte et al., 2006; Martin and Russell, 2007; Sleep et al., 2011; Sleep, 2018), where highly reducing mineralogy was likely widespread in an undifferentiated crust. The life strategies of LUCA proposed by Weiss et al. (2016) include many properties of Unc8, e.g., (1) the MAG of Unc8 harbors only CODH/ACS associated carbon fixation and energy metabolism, (2) has sodium-dependent ATPase and Mrp complex and (3) is a potential thermophile. However, the Unc8 MAG doesn’t harbor nitrogenase, hydrogenase, or superoxide dismutase. Since many studies have proposed that acetogenesis via the Wood-Ljungdahl pathway would have been a potential energy metabolism for primordial lithotrophic autotrophs (Martin and Russell, 2007; Fuchs, 2011; Lane and Martin, 2012; Martin et al., 2014), the acetogen from an analog site of early Earth may provide important insights about ancient lithotrophs and their habitats.

## Author Contributions

SS and SI designed the research and performed the analyses. SS, SI, and KN wrote the paper.

## Conflict of Interest Statement

The authors declare that the research was conducted in the absence of any commercial or financial relationships that could be construed as a potential conflict of interest.
