# Supplementary material for: Genomic and in-situ Transcriptomic Characterization of the Candidate Phylum NPL-UPL2 From Highly Alkaline Highly Reducing Serpentinized Groundwater
Source: Front Microbiol. 2018 Dec 18;9:3141. doi: 10.3389/fmicb.2018.03141 (PMC6305446; doi:10.3389/fmicb.2018.03141)
Supplement: Supplementary file 1 [file Data_Sheet_1.PDF]

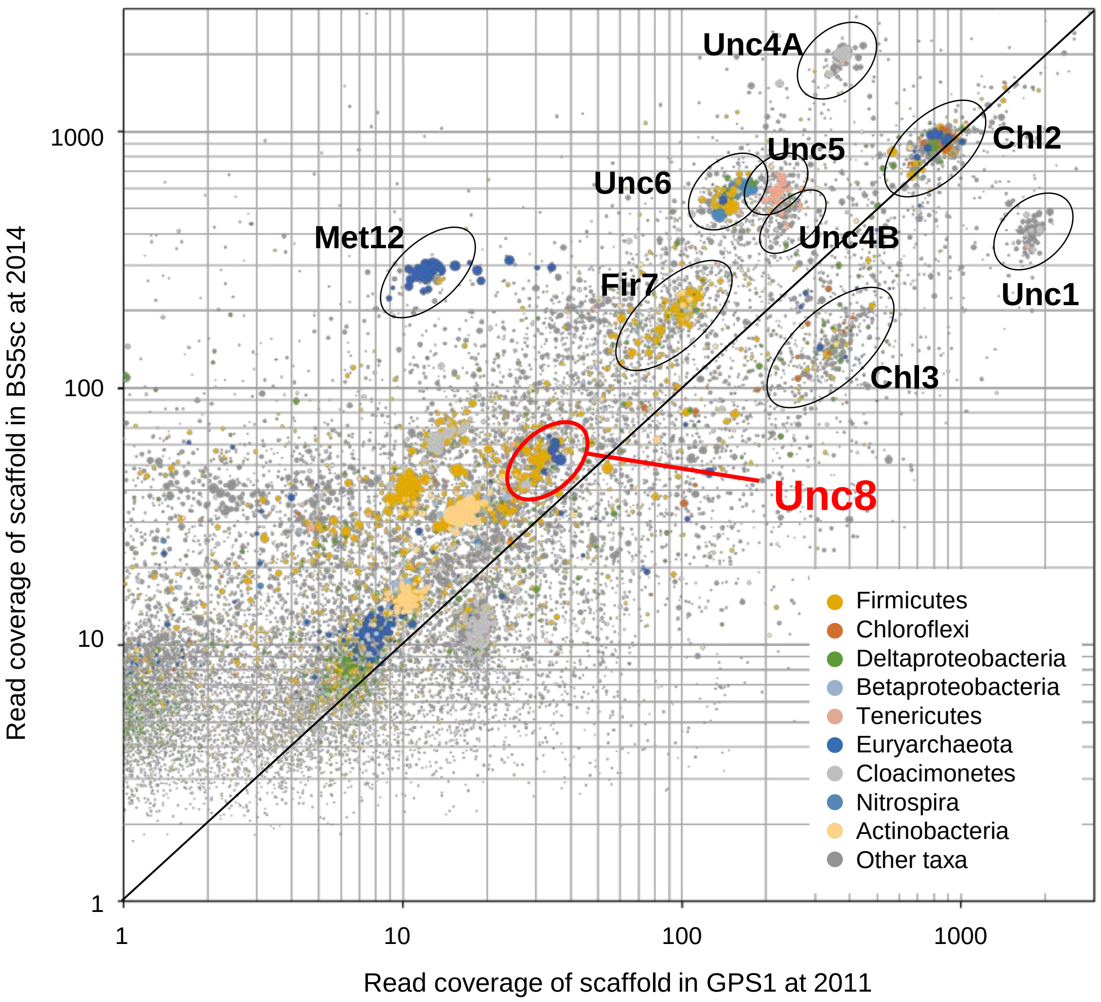

Supplementary Fig. S1 | Differential coverage plot for refining Unc8 MAG.

Unc8 was 9th – 10th dominant microbe within the communities, and the read coverage was 40-50.

A. CdhA

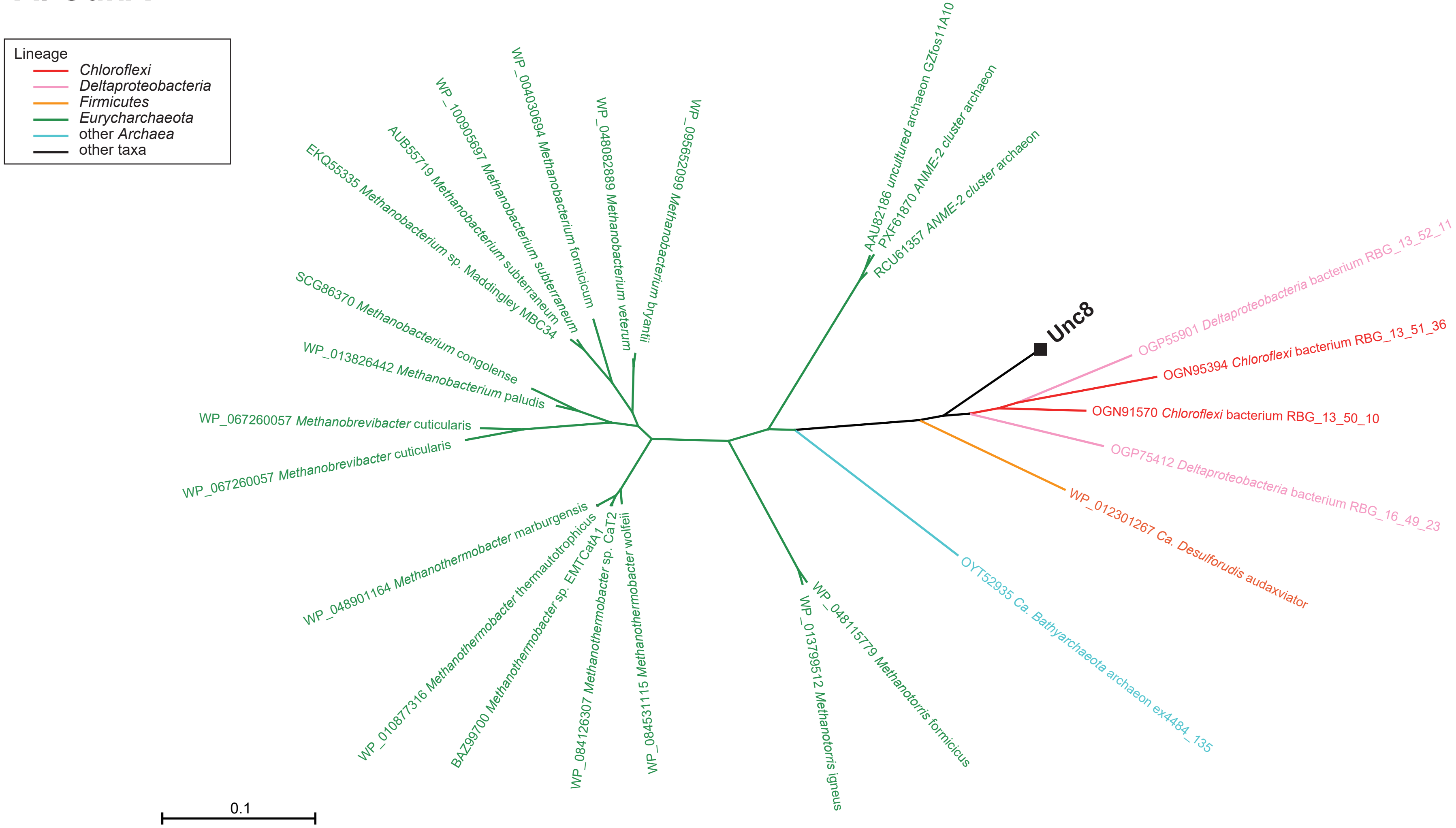

Supplementary Fig. S2 | Phylogenetic trees for CdhA (A), CdhB (B), CdhC (C), CdhD/AcsD (D), CdhE/AcsC (E), and AcdE (F).

B. CdhB

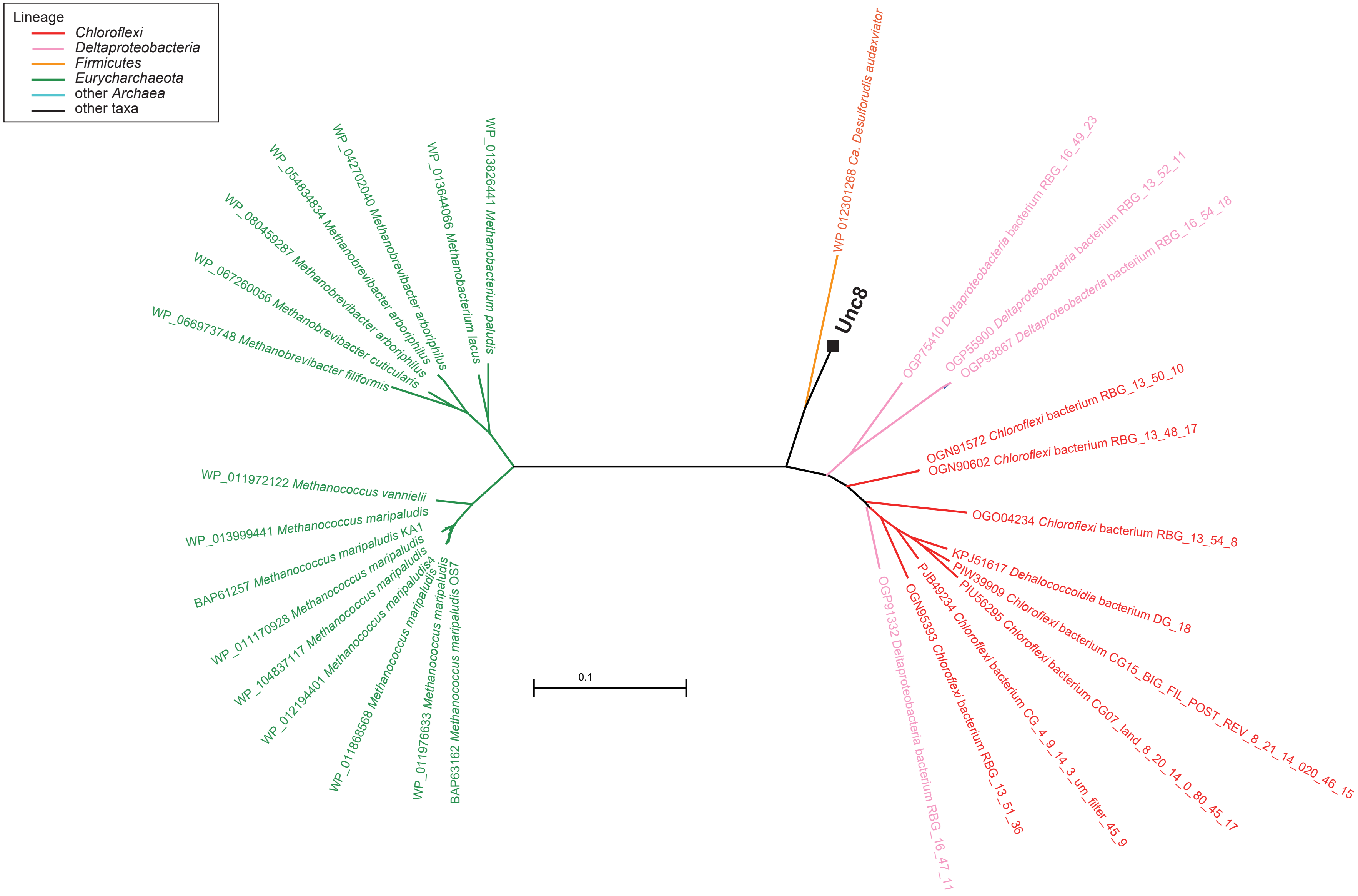
Lineage
Chloroflexi
Deltaproteobacteria
Firmicutes
Eurycharchaeota
other Archaea
other taxa
WP_012301268 Ca. Desulforudis audaxviator
Unc8
OGP75410 Deltaproteobacteria bacterium RBG_16_49_23
OGP55900 Deltaproteobacteria bacterium RBG_13_52_11
OGP93867 Deltaproteobacteria bacterium RBG_16_54_18
OGN91572 Chloroflexi bacterium RBG_13_50_10
OGN90602 Chloroflexi bacterium RBG_13_48_17
OGO04234 Chloroflexi bacterium RBG_13_54_8
KPJ51617 Dehalococcoidia bacterium DG_18
PIW39909 Chloroflexi bacterium CG15_BIG_FIL_POST_REV_8_21_14_020_46_15
PIU56295 Chloroflexi bacterium CG07_land_8_20_14_0_80_45_17
PJB49234 Chloroflexi bacterium CG_4_9_14_3_um_filter_45_9
OGN95393 Chloroflexi bacterium RBG_13_51_36
OGP91332 Deltaproteobacteria bacterium RBG_16_47_11
WP_013826441 Methanobacterium paludis
WP_013644066 Methanobacterium lacus
WP_042702040 Methanobrevibacter arboriphilus
WP_054834834 Methanobrevibacter arboriphilus
WP_080459287 Methanobrevibacter arboriphilus
WP_067260056 Methanobrevibacter cuticularis
WP_066973748 Methanobrevibacter filiformis
WP_011972122 Methanococcus vannielii
WP_013999441 Methanococcus maripaludis
BAP61257 Methanococcus maripaludis KA1
WP_011170928 Methanococcus maripaludis
WP_104837117 Methanococcus maripaludis
WP_012194401 Methanococcus maripaludis
WP_011868568 Methanococcus maripaludis
WP_011976633 Methanococcus maripaludis
BAP63162 Methanococcus maripaludis OS7
0.1

C. CdhC

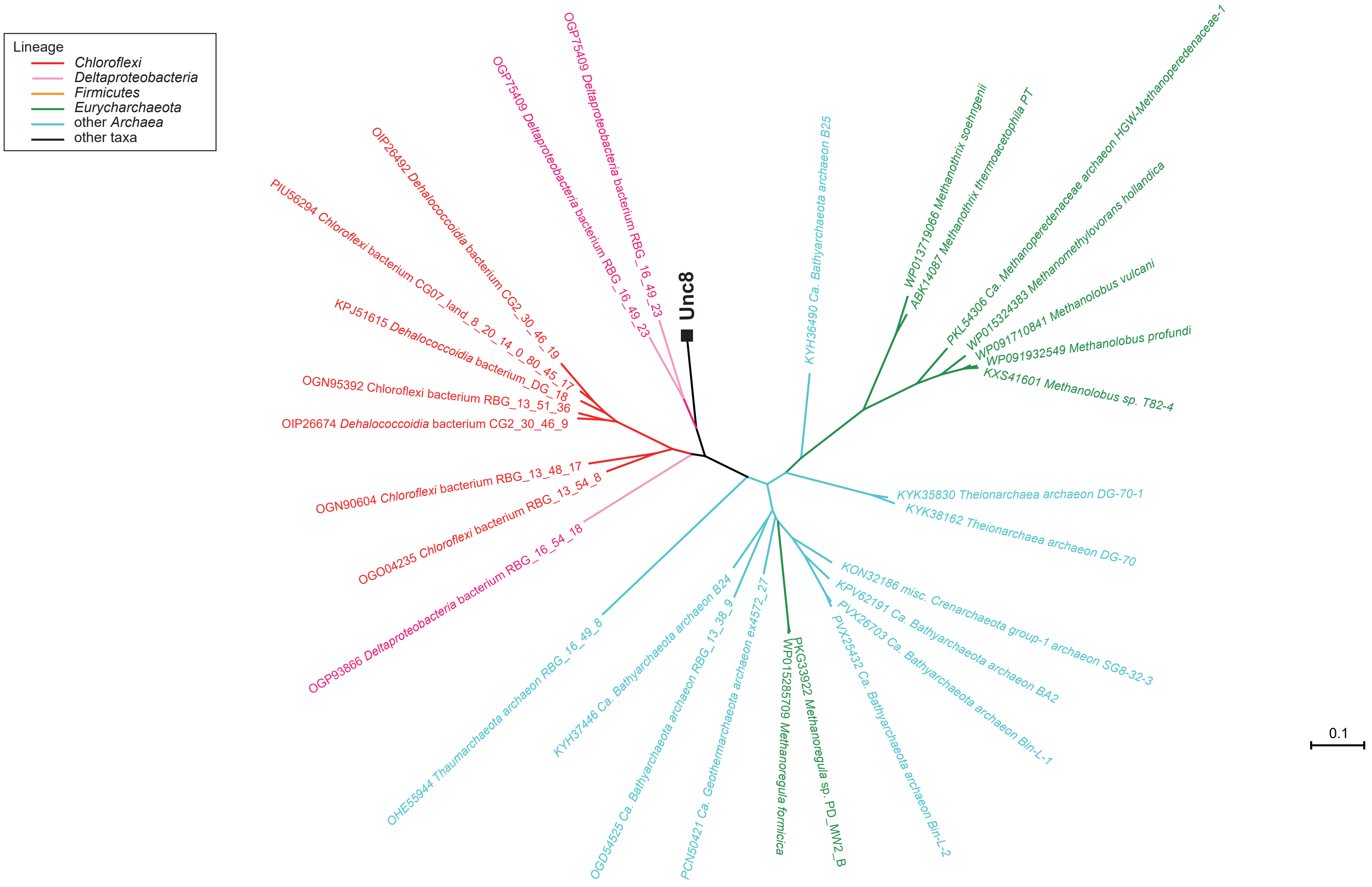
Lineage
Chloroflexi
Deltaproteobacteria
Firmicutes
Eurycharchaeota
other Archaea
other taxa
Unc8
OGP75409 Deltaproteobacteria bacterium RBG_16_49_23
OGP75409 Deltaproteobacteria bacterium RBG_16_49_23
OIP26492 Dehalococcoidia bacterium CG2_30_46_19
PIU56294 Chloroflexi bacterium CG07_land_8_20_14_0_80_45_17
KPJ51615 Dehalococcoidia bacterium_DG_18
OGN95392 Chloroflexi bacterium RBG_13_51_36
OIP26674 Dehalococcoidia bacterium CG2_30_46_9
OGN90604 Chloroflexi bacterium RBG_13_48_17
OGO04235 Chloroflexi bacterium RBG_13_54_8
OGP93866 Deltaproteobacteria bacterium RBG_16_54_18
OHE55944 Thaumarchaeota archaeon RBG_16_49_8
KYH37446 Ca. Bathyarchaeota archaeon B24
OGD54525 Ca. Bathyarchaeota archaeon RBG_13_38_9
PCN50421 Ca. Geothermarchaeota archaeon ex4572_27
WP015285709 Methanoregula formicica
PKG33922 Methanoregula sp. PD_MW2_B
PVX25432 Ca. Bathyarchaeota archaeon Bin-L-2
PVX26703 Ca. Bathyarchaeota archaeon Bin-L-1
KPV62191 Ca. Bathyarchaeota archaeon BA2
KON32186 misc. Crenarchaeota group-1 archaeon SG8-32-3
KYK38162 Theionarchaea archaeon DG-70
KYK35830 Theionarchaea archaeon DG-70-1
KYH36490 Ca. Bathyarchaeota archaeon B25
WP013719066 Methanothrix soehngenii
ABK14087 Methanothrix thermoacetophila PT
PKL54306 Ca. Methanoperedenaceae archaeon HGW-Methanoperedenaceae-1
WP015324383 Methanomethylovorans hollandica
WP091710841 Methanolobus vulcani
WP091932549 Methanolobus profundi
KXS41601 Methanolobus sp. T82-4
0.1

D. AcsD/CdhD

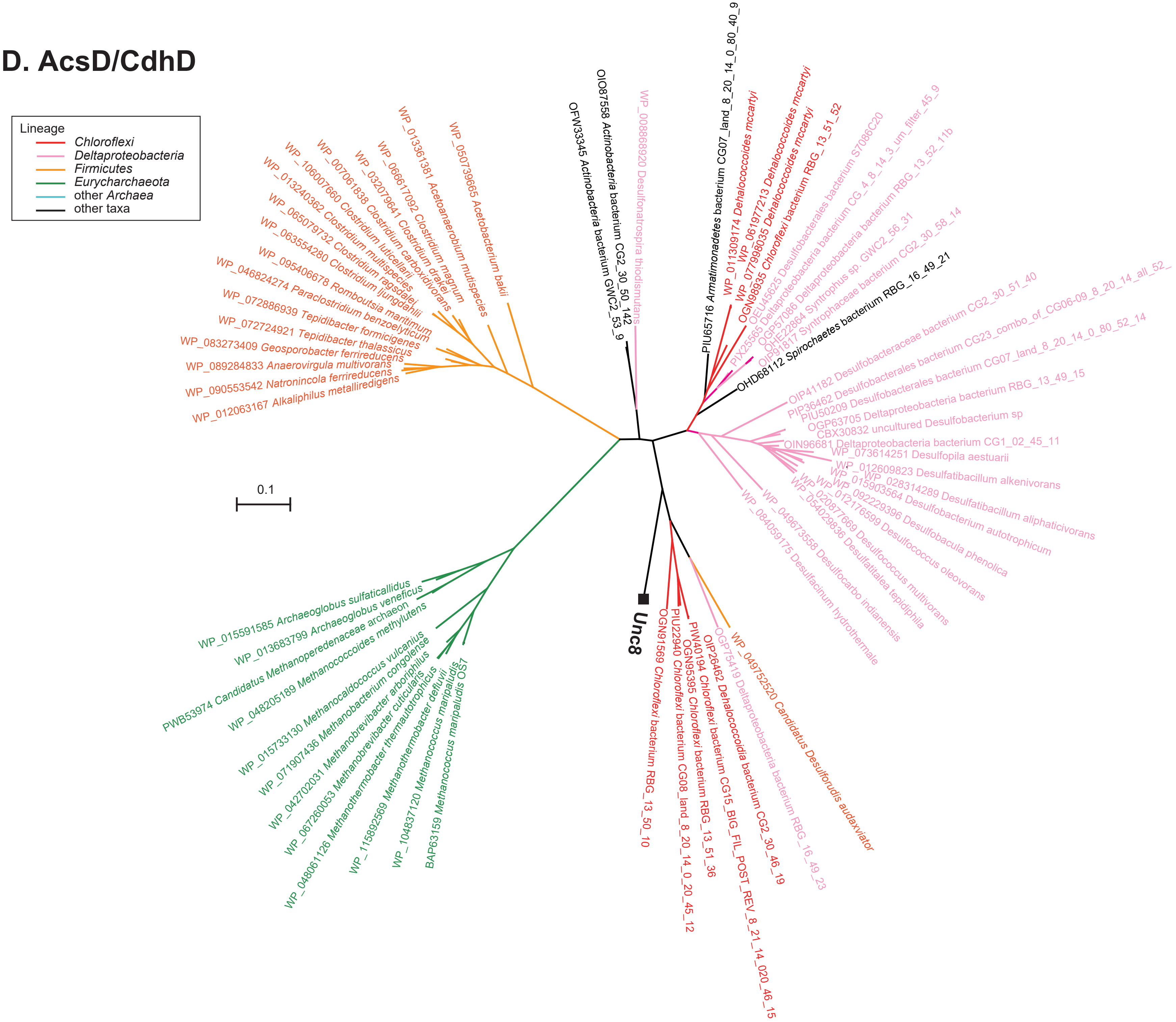

Lineage
Chloroflexi
Deltaproteobacteria
Firmicutes
Eurycharchaeota
other Archaea
other taxa
0.1
WP_050739665 Acetobacterium bakii
WP_013361381 Acetoanaerobium multispecies
WP_066617092 Clostridium magnum
WP_032079641 Clostridium carboxidivorans
WP_007061838 Clostridium drakei
WP_106007660 Clostridium luticellarii
WP_013240362 Clostridium multispecies
WP_065079732 Clostridium ragsdalei
WP_063554280 Clostridium ljungdahlii
WP_095406678 Romboutsia maritimum
WP_046824274 Paraclostridium benzoelyticum
WP_072886939 Tepidibacter formicigenes
WP_072724921 Tepidibacter thalassicus
WP_083273409 Geosporobacter ferrireducens
WP_089284833 Anaerovirgula multivorans
WP_090553542 Natronincola ferrireducens
WP_012063167 Alkaliphilus metalliredigens
OFW33345 Actinobacteria bacterium GWC2_53_9
OIO87558 Actinobacteria bacterium CG2_30_50_142
WP_008869920 Desulfonatronospira thiodismutans
PIU65716 Armatimonadetes bacterium CG07_land_8_20_14_0_80_40_9
WP_011309174 Dehalococcoides mccartyi
WP_061977213 Dehalococcoides mccartyi
WP_077998035 Dehalococcoides mccartyi
OGN98935 Chloroflexi bacterium RBG_13_51_52
OEU45625 Desulfobacterales bacterium S7086C20
PIX25565 Deltaproteobacteria bacterium CG_4_8_14_3_um_filter_45_9
OGP57086 Deltaproteobacteria bacterium RBG_13_52_11b
OHE22864 Syntrophus sp. GWC2_56_31
OIP91817 Syntrophaceae bacterium CG2_30_58_14
OHD68112 Spirochaetes bacterium RBG_16_49_21
OIP41182 Desulfobacteraceae bacterium CG2_30_51_40
PIP36462 Desulfobacterales bacterium CG23_combo_of_CG06-09_8_20_14_all_52_
PIU50209 Desulfobacterales bacterium CG07_land_8_20_14_0_80_52_14
OGP63705 Deltaproteobacteria bacterium RBG_13_49_15
CBX30832 uncultured Desulfobacterium sp
OIN96681 Deltaproteobacteria bacterium CG1_02_45_11
WP_073614251 Desulfopila aestuarii
WP_012609823 Desulfatibacillum alkenivorans
WP_028314289 Desulfatibacillum aliphaticivorans
WP_015903564 Desulfobacterium autotrophicum
WP_092229396 Desulfobacula phenolica
WP_012176599 Desulfococcus oleovorans
WP_020877669 Desulfococcus multivorans
WP_054029836 Desulfatitalea tepidiphila
WP_049673558 Desulfocarbo indianensis
WP_084059175 Desulfacinum hydrothermale
WP_049752520 Candidatus Desulforudis audaxviator
OGP75419 Deltaproteobacteria bacterium RBG_16_49_23
OIP26462 Dehalococcoidia bacterium CG2_30_46_19
PIW40194 Chloroflexi bacterium CG15_BIG_FIL_POST_REV_8_21_14_020_46_15
OGN95395 Chloroflexi bacterium RBG_13_51_36
PIU22940 Chloroflexi bacterium CG08_land_8_20_14_0_20_45_12
OGN91569 Chloroflexi bacterium RBG_13_50_10
Unc8
WP_015591585 Archaeoglobus sulfaticallidus
WP_013683799 Archaeoglobus veneficus
PWB53974 Candidatus Methanoperedenaceae archaeon
WP_048205189 Methanococcoides methylutens
WP_015733130 Methanocaldococcus vulcanius
WP_071907436 Methanobacterium congolense
WP_042702031 Methanobrevibacter arboriphilus
WP_067260053 Methanobrevibacter cuticularis
WP_048061126 Methanothermobacter thermautotrophicus
WP_115892569 Methanothermobacter defluvii
WP_104837120 Methanococcus maripaludis
BAP63159 Methanococcus maripaludis OS7

E. AcsC/CdhE

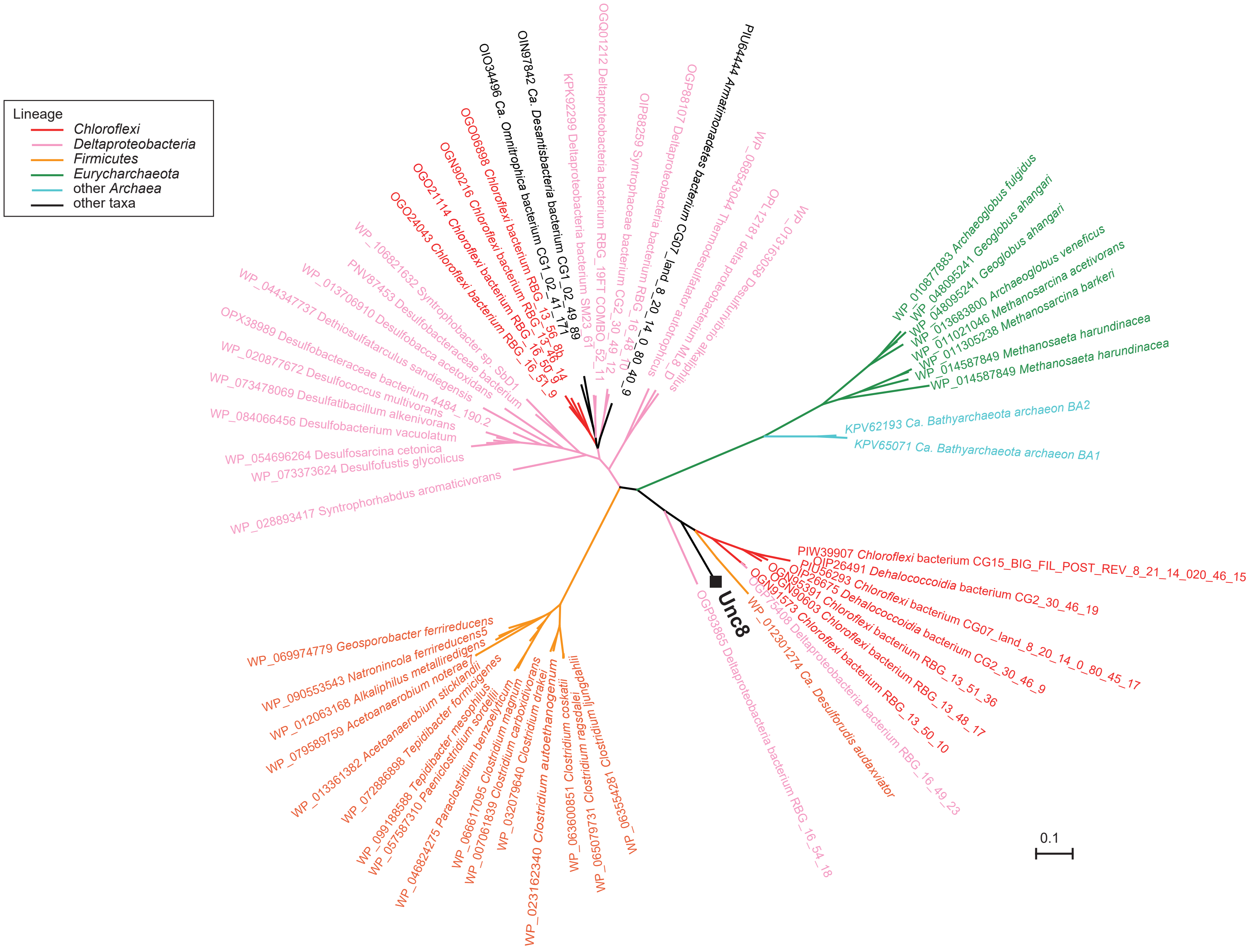

Lineage
Chloroflexi
Deltaproteobacteria
Firmicutes
Eurycharchaeota
other Archaea
other taxa
WP_106821632 Syntrophobacter sp. SbD1
PNV87453 Desulfobacteraceae bacterium
WP_013706910 Desulfobacca acetoxidans
WP_044347737 Dethiosulfatarculus sandiegensis
OPX38989 Desulfobacteraceae bacterium 4484_190.2
WP_020877672 Desulfococcus multivorans
WP_073478069 Desulfatibacillum alkenivorans
WP_084066456 Desulfobacterium vacuolatum
WP_054696264 Desulfosarcina cetonica
WP_073373624 Desulfofustis glycolicus
WP_028893417 Syntrophorhabdus aromaticivorans
OGO24043 Chloroflexi bacterium RBG_16_51_9
OGO21114 Chloroflexi bacterium RBG_16_50_9
OGN90216 Chloroflexi bacterium RBG_13_46_9
OGO06898 Chloroflexi bacterium RBG_13_56_8b_14
OIO34496 Ca. Omnitrophica bacterium CG1_02_41_171
OIN97842 Ca. Desantisbacteria bacterium CG1_02_49_89
KPK92299 Deltaproteobacteria bacterium SM23_61
OGQ01212 Deltaproteobacteria bacterium RBG_19FT_COMBO_52_11
OIP88259 Syntrophaceae bacterium CG2_30_49_12
OGP88107 Deltaproteobacteria bacterium RBG_16_48_10
PIU64444 Armatimonadetes bacterium CG07_land_8_20_14_0_80_40_9
WP_068543044 Thermodesulfatator autotrophicus
OPL12181 delta proteobacterium ML8_D
WP_013163058 Desulfurivibrio alkaliphilus
WP_010877883 Archaeoglobus fulgidus
WP_048095241 Geoglobus ahangari
WP_048095241 Geoglobus ahangari
WP_013683800 Archaeoglobus veneficus
WP_011021046 Methanosarcina acetivorans
WP_011305238 Methanosarcina barkeri
WP_014587849 Methanosaeta harundinacea
WP_014587849 Methanosaeta harundinacea
KPV62193 Ca. Bathyarchaeota archaeon BA2
KPV65071 Ca. Bathyarchaeota archaeon BA1
PIW39907 Chloroflexi bacterium CG15_BIG_FIL_POST_REV_8_21_14_020_46_15
OIP26491 Dehalococcoidia bacterium CG2_30_46_19
PIU56293 Chloroflexi bacterium CG07_land_8_20_14_0_80_45_17
OIP26675 Dehalococcoidia bacterium CG2_30_46_9
OGN95391 Chloroflexi bacterium RBG_13_51_36
OGN90603 Chloroflexi bacterium RBG_13_48_17
OGN91573 Chloroflexi bacterium RBG_13_50_10
OGP75408 Deltaproteobacteria bacterium RBG_16_49_23
WP_012301274 Ca. Desulforudis audaxviator
OGP93865 Deltaproteobacteria bacterium RBG_16_54_18
Unc8
WP_069974779 Geosporobacter ferrireducens
WP_090553543 Natronincola ferrireducens5
WP_012063168 Alkaliphilus metalliredigens
WP_079589759 Acetoanaerobium noterae
WP_013361382 Acetoanaerobium sticklandii
WP_072886898 Tepidibacter formicigenes
WP_099188588 Tepidibacter mesophilus
WP_057587310 Paeniclostridium sordellii
WP_046824275 Paraclostridium benzoelyticum
WP_066617095 Clostridium magnum
WP_007061839 Clostridium carboxidivorans
WP_032079640 Clostridium drakei
WP_023162340 Clostridium autoethanogenum
WP_063600851 Clostridium coskatii
WP_050767731 Clostridium ragsdalei
WP_063554281 Clostridium ljungdahlii
0.1

F. AcsE

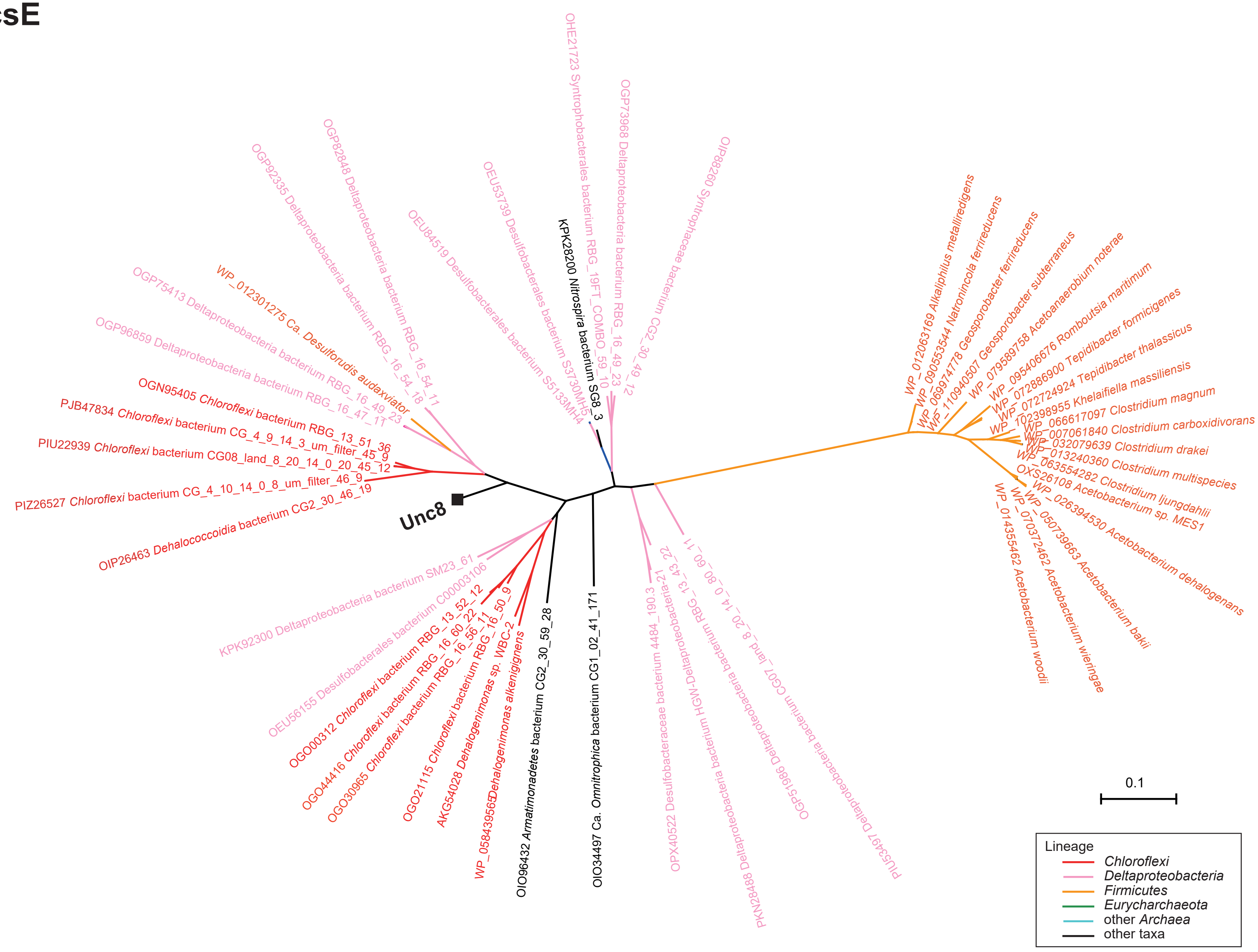
OHE21723 Syntrophobacterales bacterium RBG_19FT_COMBO_59_10
OGP73968 Deltaproteobacteria bacterium RBG_16_49_23
OIP88260 Syntrophaceae bacterium CG2_30_49_12
KPK28200 Nitrospira bacterium SG8_3
OEU53739 Desulfobacterales bacterium S3730MH5
OEU84519 Desulfobacterales bacterium S5133MH4
OGP82848 Deltaproteobacteria bacterium RBG_16_54_11
OGP92335 Deltaproteobacteria bacterium RBG_16_54_18
WP_012301275 Ca. Desulforudis audaxviator
OGP75413 Deltaproteobacteria bacterium RBG_16_49_23
OGP96859 Deltaproteobacteria bacterium RBG_16_47_11
OGN95405 Chloroflexi bacterium RBG_13_51_36
PJB47834 Chloroflexi bacterium CG_4_9_14_3_um_filter_45_9
PIU22939 Chloroflexi bacterium CG08_land_8_20_14_0_20_45_12
PIZ26527 Chloroflexi bacterium CG_4_10_14_0_8_um_filter_46_9
OIP26463 Dehalococcoidia bacterium CG2_30_46_19
Unc8
KPK92300 Deltaproteobacteria bacterium SM23_61
OEU56155 Desulfobacterales bacterium C00003106
OGO00312 Chloroflexi bacterium RBG_13_52_12
OGO44416 Chloroflexi bacterium RBG_16_60_22
OGO30965 Chloroflexi bacterium RBG_16_56_11
OGO21115 Chloroflexi bacterium RBG_16_50_9
AKG54028 Dehalogenimonas sp. WBC-2
WP_058439655 Dehalogenimonas alkenigignens
OIO96432 Armatimonadetes bacterium CG2_30_59_28
OIO34497 Ca. Omnitrophica bacterium CG1_02_41_171
OPX40522 Desulfobacteraceae bacterium 4484_190.3
PKN28488 Deltaproteobacteria bacterium HGW-Deltaproteobacteria-21
OGP51986 Deltaproteobacteria bacterium RBG_13_43_22
PIU53497 Deltaproteobacteria bacterium CG07_land_8_20_14_0_80_60_11
WP_012063169 Alkaliphilus metalliredigens
WP_090553544 Natronincola ferrireducens
WP_069974778 Geosporobacter ferrireducens
WP_110940507 Geosporobacter subterraneus
WP_079589758 Acetoanaerobium noterae
WP_095406676 Romboutsia maritimum
WP_072886900 Tepidibacter formicigenes
WP_072724924 Tepidibacter thalassicus
WP_102398955 Khelaifiella massiliensis
WP_066617097 Clostridium magnum
WP_007061840 Clostridium carboxidivorans
WP_032079639 Clostridium drakei
WP_013240360 Clostridium multispecies
WP_063554282 Clostridium ljungdahlii
OYS26108 Acetobacterium sp. MES1
WP_026394530 Acetobacterium dehalogenans
WP_050739663 Acetobacterium bakii
WP_070372462 Acetobacterium wieringae
WP_014355462 Acetobacterium woodii
0.1
Lineage
Chloroflexi
Deltaproteobacteria
Firmicutes
Eurycharchaeota
other Archaea
other taxa
